# Effects of larval exposure to sublethal doses of *Bacillus thuringiensis* var. *israelensis* on body size, oviposition and survival of adult *Anopheles coluzzii* mosquitoes

**DOI:** 10.1186/s13071-020-04132-z

**Published:** 2020-05-16

**Authors:** Steven Gowelo, James Chirombo, Jeroen Spitzen, Constantianus J. M. Koenraadt, Themba Mzilahowa, Henk van den Berg, Willem Takken, Robert McCann

**Affiliations:** 1 Laboratory of Entomology, Wageningen University & Research, Wageningen, The Netherlands; 2 Training and Research Unit of Excellence, School of Public Health, College of Medicine, Blantyre, Malawi; 3 Malawi-Liverpool-Wellcome Trust Clinical Research Programme, Blantyre, Malawi; 4 Malaria Alert Centre, College of Medicine, Blantyre, Malawi; 5 Center for Vaccine Development and Global Health, University of Maryland School of Medicine, Baltimore, USA

**Keywords:** *Bacillus thuringiensis* var. *israelensis*, Sublethal dose, Larval source management, Mosquito, Vector control

## Abstract

**Background:**

Application of the larvicide *Bacillus thuringiensis* var. *israelensis* (*Bti*) is a viable complementary strategy for malaria control. Efficacy of *Bti* is dose-dependent. There is a knowledge gap on the effects of larval exposure to sublethal *Bti* doses on emerging adult mosquitoes. The present study examined the effect of larval exposure to sublethal doses of *Bti* on the survival, body size and oviposition rate in adult *Anopheles coluzzii*.

**Methods:**

Third-instar *An. coluzzii* larvae were exposed to control and sublethal *Bti* concentrations at LC_20_, LC_50_ and LC_70_ for 48 h. Surviving larvae were reared to adults under standard colony conditions. Thirty randomly selected females from each treatment were placed in separate cages and allowed to blood feed. Twenty-five gravid females from the blood-feeding cages were randomly selected and transferred into new cages where they were provided with oviposition cups. Numbers of eggs laid in each cage and mortality of all adult mosquitoes were recorded daily. Wing lengths were measured of 570 mosquitoes as a proxy for body size.

**Results:**

Exposure to LC_70_*Bti* doses for 48 h as third-instar larvae reduced longevity of adult *An. coluzzii* mosquitoes. Time to death was 2.58 times shorter in females exposed to LC_70_*Bti* when compared to the control females. Estimated mortality hazard rates were also higher in females exposed to the LC_50_ and LC_20_ treatments, but these differences were not statistically significant. The females exposed to LC_70_ concentrations had 12% longer wings than the control group (*P* < 0.01). No differences in oviposition rate of the gravid females were observed between the treatments.

**Conclusions:**

Exposure of *An. coluzzii* larvae to sublethal *Bti* doses reduces longevity of resultant adults and is associated with larger adult size and unclear effect on oviposition. These findings suggest that anopheline larval exposure to sublethal *Bti* doses, though not recommended, could reduce vectorial capacity for malaria vector populations by increasing mortality of resultant adults.
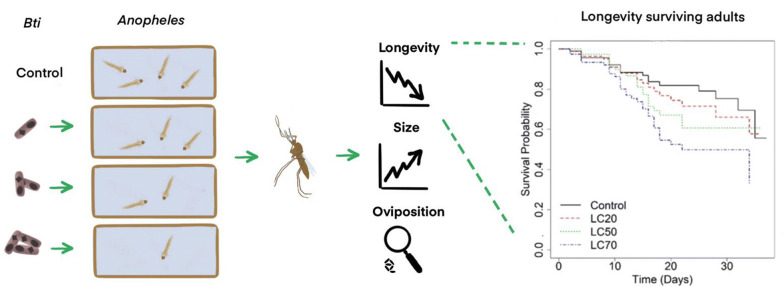

## Background

Malaria remains a major public health problem in the world, especially in Africa where the incidence rate has remained stable, and in some cases even increased, over the past few years [1] despite widespread use of control methods [2]. Widespread insecticide resistance has been reported in the important malaria vectors across all classes of insecticides used on insecticide-treated bednets and indoor residual spraying [3–6]. Further, outdoor feeding and resting by malaria vectors, whether in response to use of insecticide-treated bednets [7–10] or as a natural behaviour in some vector populations, expose people to residual malaria transmission. This has led to global advocacy for additional vector control tools to eliminate residual malaria transmission by targeting outdoor resting and feeding, as well as insecticide-resistant vectors [9]. Larval source management (LSM) indiscriminately kills malaria vectors before they emerge as adult mosquitoes [11] and relies on separate modes of action from those used in insecticide-treated bednets and indoor residual spraying. Thus, LSM can contribute to malaria control where the vectors exhibit exophagic and exophilic behaviours, and in settings where insecticide resistance has emerged.

Strains of the bacteria *Bacillus thuringiensis* var. *Israelensis* (*Bti*) are widely used as active ingredients in larvicides because they do not cause harm to non-target organisms or the environment [12, 13]. The bacteria produce parasporal crystalline protein inclusions (Cry and Cyt) which are lethal only to specific insect taxa [14]. In mosquitoes, the protein crystals bind to specific receptors exposed on the surface of the plasma membrane and then insert into the membrane, creating lytic pores in microvilli of apical membranes [15] that disturb the cell’s osmotic balance, resulting in cell lysis and consequently death of the larvae [16, 17]. The low persistence of *Bti* toxins under field conditions [18] makes *Bti* an eco-friendly larvicide even when used in repeated treatments from three to seven years [19]. This rapid degradation, however, necessitates repeated applications of the larvicide for effective control of target organisms.

Environmental conditions experienced by mosquitoes during larval growth and development affect adult fitness in a number of ways [20–22]. Nutritional and larvicidal stresses can reduce adult size, survival and fecundity in different mosquito species [23–26]. One such larvicidal stressor could be sublethal doses of *Bti*. Sublethal *Bti* concentrations reduce adult mosquito survival rates, lower blood-engorgement rate and egg production, increase development time from egg to adult, and decrease offspring sex ratio in *Aedes aegypti* [27]. Similarly, sublethal doses of *Bti* may cause adverse effects on life parameters of exposed *Ae. aegypti* and their unexposed first-generation progeny [26]. Prolonged development time, reduced longevity and reduced reproductive rates were observed in *Anopheles superpictus* exposed to sublethal doses of *Bti* [28].

Mosquito larvae may be exposed to sublethal concentrations of *Bti* under field conditions. This could result from some or all of these factors: (i) anthropological factors such as poor measurement of *Bti* in relation to habitat size and poor calibration of instruments used in weighing the product; (ii) biotic factors such as growth of vegetation in habitats that may trap the product and reduce larva-product contact; and (iii) abiotic factors such as water pH, turbidity and temperature, which may degrade the product or modulate product activity within the mosquito. These doses may result directly or indirectly in biological changes in the surviving larvae and consequently impact adult fitness. Though not ideal, sublethal *Bti* doses could impact vector populations and malaria parasite transmission, and should thus be well understood. The purpose of this study was to understand how these doses affect the survival, body size and oviposition rate of *Anopheles coluzzii*, an important human malaria vector in Africa.

## Methods

### *Anopheles coluzzii* colony

The mosquitoes used in all experiments came from an *An. coluzzii* colony maintained at the insectary of the Laboratory of Entomology of Wageningen University, The Netherlands. Standard colony rearing conditions for the immature stages consisted of plastic larval trays (10 × 25 × 8 cm) filled with salt-treated demineralised water (0.008 g/ml) to reduce the potential for larval pathogen infections. In each start-up tray, salt-treated water containing approximately 200 first-instar larvae was pipetted. Larvae were provided 0.1 mg/larva Tetramin fish food (Tetrawerke, Melle, Germany) for the first instars and 0.3 mg/larva for the other larval stages. All pupae were removed from the trays and placed in salt-treated demineralised water in 100 ml plastic cups in 30 × 30 × 30 cm cages. Emerging adults were fed 6% sugar solution *ad libitum*. From day 3–4 post-emergence, adult females were offered human blood for 2–3 h per day for 11 days. A membrane feeding system (Discovery Workshops, Acrington, Lancs, UK) [29] was used for the blood-feeding.

### Sublethal *Bti* concentration determination

The protocol of Becker and Rettich [30] was adopted for preparing a *Bti* solution. Fifty milligrams of a water-dispersible granule (WDG) formulation of *Bti* (VectoBac® WDG, Valent BioSciences) were added to 10 ml distilled water. The mixture was homogenised at 700 *rpm* for 10 min and vortexed for 15 min. Then 1 ml of the homogenised suspension was added to 49 ml distilled water to make a stock solution of 100 mg/l. The suspension was vortexed for 5 s at maximum speed. Aliquots of the suspension ranging from 0 to 1200 µl were pipetted into plastic cups containing 100 ml salt-treated demineralised water to produce final experimental concentrations ranging from 0 to 0.4 mg/l (Additional file 1: Table S1).

Over six different days, larvae were exposed to three treatment cups per concentration and three control cups (salt-treated demineralised water). Twenty-five third-instar larvae were placed in each cup, and mortality was recorded after 24 and 48 h. If pupation occurred, the pupae were removed, and their numbers were excluded from calculations. Based on the proportion of larval mortality observed at each *Bti* concentration after 48 h, concentrations producing about 20% mortality (LC_20_), 50% mortality (LC_50_) and 70% mortality (LC_70_) were fixed and used throughout the subsequent experiments.

### Tests of sublethal doses on fitness parameters

To determine the effects of sublethal exposure to *Bti* during larval development, trays with third-instar larvae were obtained from the insectary and were exposed to experimental conditions for 48 h. These experiments were replicated six times. For each replicate, we used three control trays containing the salt-treated demineralised water and three trays for each of the three sublethal *Bti* concentrations (LC_20_, LC_50_ and LC_70_). During the 48-h exposure period, each tray was provided 20 mg fish food per day. Air temperature in the climate chamber was maintained at 27 ± 1 °C. All dead and moribund larvae were counted after 24 and 48 h. After the 48-h exposure period for each replicate, all surviving larvae from the same treatment were pooled and placed in new trays with fresh salt-treated water only (i.e. no *Bti*). Sixty milligrams of fish food were given to the larvae in each tray daily. All emerging pupae were placed in plastic cups (100 × 50 mm diameter), which were placed in 30 × 30 × 30 cm cages, separated by treatment and replicate. The emerging adults were fed 6% sugar solution *ad libitum*. On days 5 and 6 post-emergence, 30 females from each treatment were indiscriminately removed using a mouth aspirator and placed in new cages, still separated by treatment and replicate. The females in each cage were given a chance to feed on human blood *via* arm feeding for 10 min per day for two days. The same person was used for all the blood-feeding. The mosquitoes were also provided with 6% sugar solution in the new cages when not blood-feeding. At 24 h after blood-feeding, oviposition cups were introduced in the cages and 25 randomly selected gravid females were kept in the cage. The other five females were taken back to the non-oviposition cages which contained mosquitoes which were not given blood meals. All dead mosquitoes from day of emergence in both oviposition and non-oviposition cages were counted daily until 37 days post-emergence. The records were separated by treatment, replicate and sex. The number of eggs laid was counted daily for seven days and separated by treatment and replicate. Wing lengths were measured of 570 mosquitoes as the distance between the alula and the wing tip, excluding fringe scales, using CMEX DC 5000 binocular microscope (Euromex, The Netherlands) and Image Focus Version 3 software. The wing measurements were separated by treatment, replicate and sex of the mosquitoes.

### Data analysis

To determine the impact of *Bti* concentration on wing length, we fitted a linear mixed model to account for the effect of treatment (as a fixed effect) and replicate (as a random effect). We fitted a random intercept model where the effect of replicate was allowed to deviate from the overall, to investigate if replicate as a covariate contributed to the overall variation in the wing length. Kaplan Meier curves were plotted to visualize mosquito survival patterns over time. To estimate the hazard rate of mortality for each level of *Bti* concentration, we fitted a multivariate Cox proportional hazards model with treatment and replicate as covariates. A Poisson generalized linear model with treatment and wing length as covariates was fitted to investigate their effect on mean number of eggs laid by gravid females. The level of significance was set at 0.05. All statistical analyses were carried out in R version 3.6.1.

## Results

### Determination of sublethal concentrations of *Bti* on *An. coluzzii* larvae

**Fig. 1:**
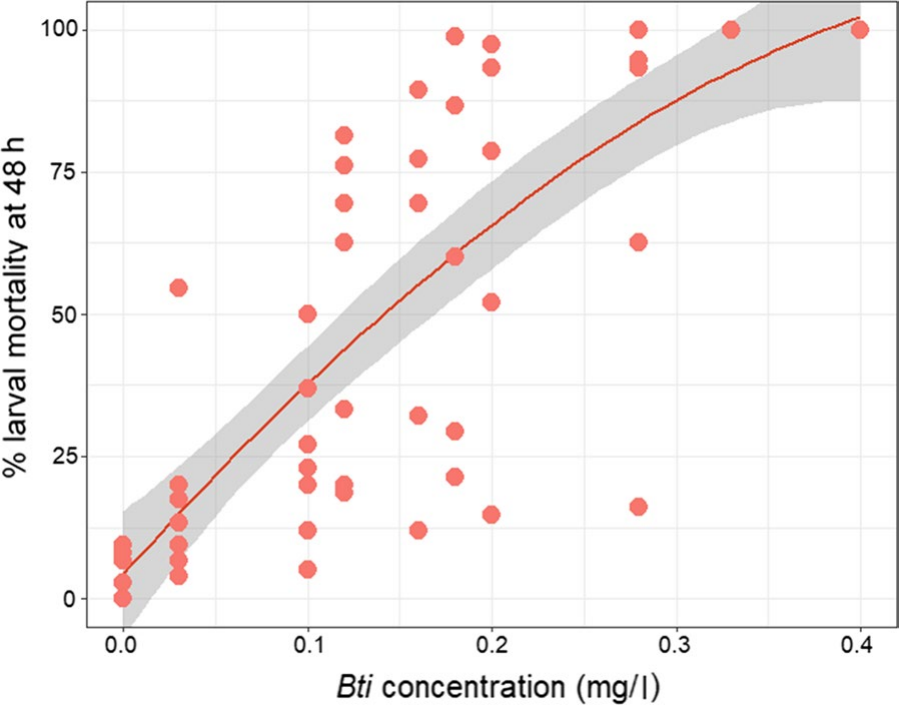
The relationship between *Bti* concentration and mortality of *Anopheles coluzzii* larvae after 48 h. Each point represents the *Bti* concentration and corresponding larval mortality in each cup per replicate. Overlapping points indicate the same values for multiple cups. The red line is the weighted mean larval mortality due to the varied *Bti* concentrations and the grey area represents the 95% confidence interval. See Additional file 1: Table S1 for the number of replicates run for each *Bti* concentration

Mortality of *An. coluzzii* larvae exposed as third instars to *Bti* for 48 h increased with increasing *Bti* concentrations (Fig. 1). Based on these larval mortality rates, we selected 0.03 mg/l, 0.12 mg/l and 0.28 mg/l as *Bti* concentrations for LC_20_, LC_50_ and LC_70_ respectively (Additional file 1: Table S1).

### Effect of larval exposure to sublethal *Bti* on post-larval stage counts in *An. coluzzii*

A total of 2902 adult mosquitoes emerged from larvae from the control and treatment trays. The number of mosquitoes surviving to the adult stage decreased with increasing *Bti* concentrations (Table 1). In the control groups, there were 679 (69.9%) females and 293 (30.1%) males. For the treatment groups, 524 (60.8%) and 337 (39.1%), 423 (66.7%) and 211 (33.3%), and 315 (72.4%) and 120 (27.6%) were females and males for LC_20_, LC5_0_ and LC_70_, respectively. Treatment did not have an effect on the sex ratio of mosquitoes surviving to the adult stage (*χ*^2^ = 0, *df* = 3, *P* = 1).

**Table 1:** Effect of larval exposure to sublethal *Bti* on post-larval stages in *An. coluzzii*

Treatment	Mean no. of pupae ± SE	Mean no. of adults ± SE	Mean wing length (mm) of adult males ± SE (*n*)	Mean wing length (mm) of adult females ± SE (*n*)
Control	162.0 ± 9.02	159 ± 7.33	2.39 ± 0.02 (57)	2.36 ± 0.02 (160)
LC_20_	143.5 ± 13.68	137 ± 9.04	2.36 ± 0.03 (40)	2.41 ± 0.02 (133)
LC_50_	105.7 ± 13.63	93 ± 10.23	2.42 ± 0.04 (37)	2.50 ± 0.02 (63)
LC_70_	72.5 ± 7.98	64 ± 5.32	2.48 ± 0.02* (34)	2.58 ± 0.05* (46)

*Indicates statistical significance of the treatment in relation to the control (p<0.01). See Additional file 1: Tables S2 and S3 for the effect sizes of larval exposure to the sublethal *Bti* concentrations on wing lengths of adult males and females, respectively

### Effect of larval exposure to sublethal *Bti* concentrations on wing length

Five hundred and seventy wings were measured and separated by sex: 402 and 168 for female and male mosquitoes, respectively. For both sexes, increasing *Bti* concentration was associated with an increase in wing length (Table 1), but the difference was only significant between the control and LC_70_. The mean wing length of the adult female *An. coluzzii* exposed to LC_70_*Bti* concentrations increased by 12% compared to the control group (Additional file 1: Table S2). Similarly, adult *An. coluzzii* males exposed to LC_70_ concentrations had wings that were 20% longer than the control group (Additional file 1: Table S3).

### Survival of adult *An. coluzzii* after exposure to sublethal *Bti* concentrations as larvae

**Fig. 2:**
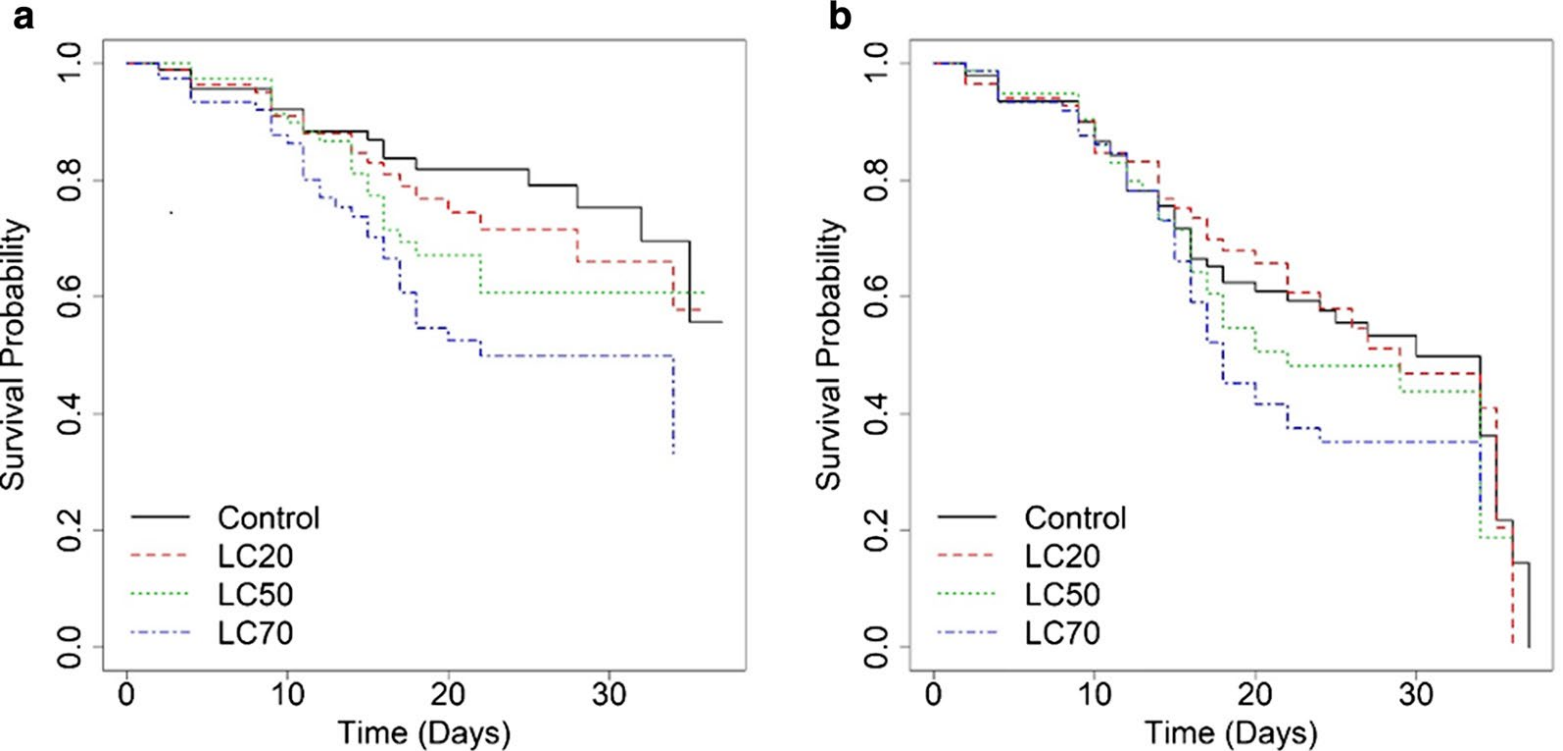
Survival curves for *Anopheles coluzzii* adults exposed to different concentrations of *Bti* as larvae. **a** Survival curves for adult female *An. coluzzii.***b** Survival curves for adult male *An. coluzzii*

Survival of adult *An. coluzzii* decreased with increasing *Bti* concentration exposure as larvae (Fig. 2). The highest cumulative survival probabilities for both female and male mosquitoes were observed in the control and LC_20_ concentrations. The survival probabilities dropped more rapidly in males compared to females.

An increasing trend of hazard ratios (HR) with increasing *Bti* concentrations was observed in adult females compared with the control group (Table 2). When exposed to *Bti* LC_70_ as larvae, the proportional hazard rate for mortality as adult females was about three times higher than the rate from the control group (HR = 2.58, CI: 1.44–4.53, *P* < 0.01). The mortality hazard ratio for adult males was also significantly increased when exposed to *Bti* LC70 as larvae, (HR =1.54, CI: 0.99–2.38, *P* = 0.049; Table 2).

**Table 2:** Mortality of adult *An. coluzzii* exposed to sublethal *Bti* concentrastions as larvae

Variable	Time to death (females)			Time to death (males)		
	HR	95% CI	*P*-value	HR	95% CI	*P*-value
LC_20_	1.25	0.65–2.38	0.5	0.95	0.6–1.51	0.83
LC_50_	1.62	0.86–3.05	0.14	1.25	0.8–1.96	0.33
LC_70_	2.58	1.44–4.63	**0.001**	1.54	0.99–2.38	**0.049**

*Note*: Bold values indicate statistical significance of the treatment in relation to the control (p<0.05)

### Effect of sublethal *Bti* concentrations on number of eggs laid by adult *An. coluzzii*

**Fig. 3:**
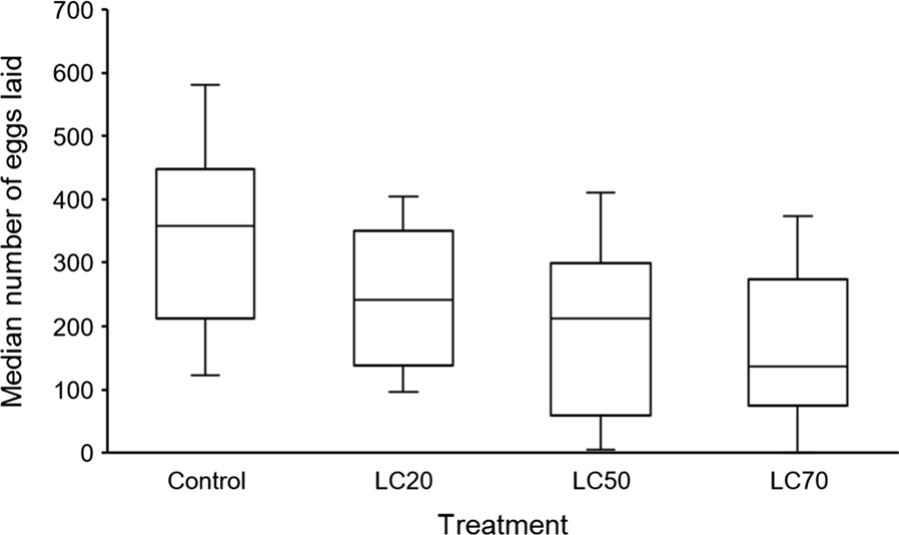
Effect of sublethal *Bti* doses on the median number of eggs laid by a group of 25 gravid females. The 25-female egg count was repeated 6 times within each treatment. No statistical differences were observed (Table 3)

There was an apparent trend of decreasing number of eggs laid per cage of 25 females with increasing concentration of *Bti* exposure as larvae (Fig. 3). However, these differences were not statistically significant (Table 3). Further, the mean wing length of gravid females per cage did not have an effect on the number of eggs laid (Table 3).

**Table 3:** Effect of sublethal *Bti* and wing length on egg-laying in female *An. coluzzii*

Variable	Estimate	95% CI	*P*-value
Intercept	1.16	0.59–2.26	0.667
LC_20_	0.72	0.41–1.26	0.55
LC_50_	0.58	0.27–1.24	0.32
LC_70_	0.52	0.23–1.13	0.25
Mean wing length	0.75	0.06–5.45	0.72

## Discussion

This study assessed potential effects of larval exposure to sublethal doses of *Bti* on fitness parameters of adult *An. coluzzii.* We observed that larval exposure to sublethal *Bti* doses reduced survival of adult *An. coluzzii* mosquitoes. For both male and female *An. coluzzii*, the lowest survival probability was recorded at the highest *Bti* concentration. It is not well understood how the larvicide reduces longevity of the adults that survive exposure as larvae. However, there is evidence that a *Bti* toxin, Cry1C, has a toxic effect to brain cells of larvae of a lepidopteran, *Lymantria dispar*, *in vitro* [31]. This suggests that *Bti* may cause similar damage to the adult mosquitoes surviving larval exposure to sublethal doses which may reduce their life spans. Similar results have been reported for *Culex quinquefasciatus* exposed to sublethal doses of cypermethrin as both larvae and adults [32]. The authors attributed their findings to physiological damage caused to the nervous system and associated aberrations due to abnormal hormone release and dehydration as a result of exposure to cypermethrin. Malaria parasites require 8–35 days to develop in their anopheline hosts [33], and therefore reduced adult longevity is widely recognized to reduce the vectorial capacity of a vector population [34]. Under highly controlled laboratory conditions, 10% and 37% female mortalities were observed by day 15 in the control and LC_70_ groups, respectively, suggesting that the sublethal doses can potentially contribute to reductions in malaria parasite transmission by reducing the longevity of adults.

Wing length in adult *An. coluzzii* mosquitoes increased with larval exposure to increasing sublethal *Bti* concentrations. Similar findings were observed in adult female *Ae. aegypti* mosquitoes exposed to sublethal concentrations of a naturally derived insecticide, spinosad [35]. The larger mosquitoes emerging from larval development at higher sublethal *Bti* concentrations may have at least two explanations. First, larger mosquito larvae may be more capable of coping with any stress induced by *Bti* exposure, and therefore survived exposure to concentrations that smaller larvae could not. Secondly, *Bti* treatment reduced larval densities and, thus, competition over food and other resources following *Bti* exposure. Reduced resource competition due to lower larval densities has previously been associated with larger mosquito size [36, 37]. Wing length is used as a standard indicator of body size in mosquitoes [38, 39] as the two measures are positively correlated. Larger females typically produce more eggs because they can take larger blood meals, which means they contribute more offspring to the population [40, 41]. Larger size has also been related with better ability to disperse in *Culex pipiens* [42]. The potential increase in oviposition rates and dispersal ability for larger mosquitoes may increase their contribution to malaria transmission. Additionally, larger mosquitoes may exhibit reduced susceptibility to the synthetic insecticides used in current vector control tools [43, 44], although it is unclear how this effect might interact with the reduced survival of *An. coluzzii* exposed to sublethal concentrations of *Bti* observed in our study. It is also known that smaller female mosquitoes require multiple blood meals before they can reproduce, thus increasing their contact with hosts and effectively becoming more efficient vectors [37]. Therefore, the impact of larger adult *An. coluzzii* mosquitoes on malaria parasite transmission due to sublethal *Bti* concentrations remains unclear.

We observed no associations between the mean number of eggs laid and *Bti* treatment. Similar findings have been observed in another malaria vector, *An. superpictus*, also exposed to sublethal concentrations of *Bti* as larvae under laboratory conditions [28]. Also, the results agree with observations made on *Ae. aegypti* under similar conditions [26]. Our study might have been limited by clustering of the 25 gravid females in an oviposition cage containing only one oviposition cup. Clustering of females in a cage might have led to egg retention in some of the females as a way of avoiding competition for oviposition space. Evidence of egg retention has been reported in gravid female mosquitoes in absence of suitable oviposition sites [45]. The mean wing length of gravid females also was not associated with the number of eggs laid per cage. The averaging of wing lengths of gravid females might have masked small but meaningful variations in wing sizes due to treatments. Despite not significantly explaining differences in the egg counts between control and treatment groups, we observed a declining trend in median number of eggs laid with increasing *Bti* concentrations. This effect deserves further study, as a reduction in the number of eggs with larval exposure to *Bti* would directly reduce vector population size.

## Conclusions

Exposure of *An. coluzzii* larvae to sublethal *Bti* doses reduced longevity of adult *An. coluzzii* and was associated with larger adult size. Whether the increased size is mechanistically linked to *Bti* toxins or decreased larval density is unclear. There was not a clear effect of larval exposure to *Bti* on oviposition. It remains important to apply the recommended dosage when applying *Bti* for malaria vector control, as concentrations high enough to kill larvae before they emerge as adults provide the most effective control against malaria parasite transmission. Still, the effect of sublethal *Bti* exposure could lead to a reduction in vectorial capacity for malaria vector populations by increasing mortality of adults that survived exposure to *Bti* in their larval stage.

## Supplementary information

**Additional file 1.** Additional tables.

## Data Availability

Data supporting the conclusions of this article are included within the article. The datasets used and/or analysed during the present study are available from the corresponding author upon reasonable request.

## Abbreviations

*Bti*: *Bacillus thuringiensis israelensis*
WDG: water-dispersible granule
LSM: larval source management
LC: lethal concentration
