# Supplementary material for: Effects of larval exposure to sublethal doses of Bacillus thuringiensis var. israelensis on body size, oviposition and survival of adult Anopheles coluzzii mosquitoes
Source: Parasit Vectors. 2020 May 16;13:259. doi: 10.1186/s13071-020-04132-z (PMC7229702; doi:10.1186/s13071-020-04132-z)
Supplement: Supplementary file 1 — Additional file 1. Additional tables. [file 13071_2020_4132_MOESM1_ESM.docx]

**Additional file 1: Table S1.** Concentrations of *Bti* used in the bioassay determination experiments

| **Volume (ul) *Bti* 1ml:49ml stock solution** | **Concentration of *Bti* (mg/L)** | **Number of replicates** | **Mean number of dead larvae per tray* ± SE** | **Mean % mortality*** |
| --- | --- | --- | --- | --- |
| 0 | 0 | 7 | 3.2 ± 2.1 | 4.3 |
| 90 | 0.03 | 7 | 13.4 ± 8.6 | 17.9 |
| 300 | 0.1 | 7 | 32.4 ± 4.5 | 24.9 |
| 360 | 0.12 | 7 | 38.7 ± 3.1 | 51.7 |
| 480 | 0.16 | 6 | 30 ± 3.7 | 56 |
| 540 | 0.18 | 6 | 44.4 ± 4.4 | 59.2 |
| 600 | 0.2 | 6 | 50.4 ± 3.9 | 67.2 |
| 840 | 0.28 | 6 | 55 ± 3.7 | 73.3 |
| 1000 | 0.33 | 6 | 75 ± 0 | 100 |
| 1200 | 0.4 | 6 | 75 ± 0 | 100 |

Mortality was observed of *An. coluzzii* larvae at each concentration after starting with 75 larvae across 3 cups per treatment per replicate. Preparation of the stock solution is explained in the methods section. *across replicates after 48hrs

**Additional file 1: Table S2.** Effect of larval exposure to sublethal *Bti* on female *An. coluzzii* wing lengths*.*

| **Variable** | **Estimate** | **SE** | **P-value** |
| --- | --- | --- | --- |
| Intercept | 2.42 | 0.032 | **<0.01** |
| LC20 | -0.02 | 0.034 | 0.62 |
| LC50 | 0.04 | 0.035 | 0.24 |
| LC70 | 0.12 | 0.035 | **<0.01** |

Bold values indicate statistical significance of the treatment in relation to the control (p<0.01).

**Additional file 1: Table S3.** Effect of larval exposure to sublethal *Bti* on male *An. coluzzii* wing lengths*.*

| **Variable** | **Estimate** | **SE** | **P-value** |
| --- | --- | --- | --- |
| Intercept | 2.36 | 0.03 | **<0.01** |
| LC20 | 0 | 0.05 | 0.97 |
| LC50 | 0.05 | 0.04 | 0.29 |
| LC70 | 0.2 | 0.05 | **<0.01** |

Bold values indicate statistical significance of the treatment in relation to the control (p<0.05).
